# An Improved Genotyping by Sequencing (GBS) Approach Offering Increased Versatility and Efficiency of SNP Discovery and Genotyping

**DOI:** 10.1371/journal.pone.0054603

**Published:** 2013-01-23

**Authors:** Humira Sonah, Maxime Bastien, Elmer Iquira, Aurélie Tardivel, Gaétan Légaré, Brian Boyle, Éric Normandeau, Jérôme Laroche, Stéphane Larose, Martine Jean, François Belzile

**Affiliations:** 1 Département de Phytologie and Institut de biologie intégrative et des systèmes, Université Laval, Quebec City, Quebec, Canada; 2 Plateforme d’analyses génomiques and Institut de biologie intégrative et des systèmes, Université Laval, Quebec City, Quebec, Canada; 3 Département de Biologie, and Institut de biologie intégrative et des systèmes, Université Laval, Quebec City, Quebec, Canada; 4 Plate-forme de bio-informatique and Institut de biologie intégrative et des systèmes, Université Laval, Quebec City, Quebec, Canada; Auburn University, United States of America

## Abstract

Highly parallel SNP genotyping platforms have been developed for some important crop species, but these platforms typically carry a high cost per sample for first-time or small-scale users. In contrast, recently developed genotyping by sequencing (GBS) approaches offer a highly cost effective alternative for simultaneous SNP discovery and genotyping. In the present investigation, we have explored the use of GBS in soybean. In addition to developing a novel analysis pipeline to call SNPs and indels from the resulting sequence reads, we have devised a modified library preparation protocol to alter the degree of complexity reduction. We used a set of eight diverse soybean genotypes to conduct a pilot scale test of the protocol and pipeline. Using *Ape*KI for GBS library preparation and sequencing on an Illumina GAIIx machine, we obtained 5.5 M reads and these were processed using our pipeline. A total of 10,120 high quality SNPs were obtained and the distribution of these SNPs mirrored closely the distribution of gene-rich regions in the soybean genome. A total of 39.5% of the SNPs were present in genic regions and 52.5% of these were located in the coding sequence. Validation of over 400 genotypes at a set of randomly selected SNPs using Sanger sequencing showed a 98% success rate. We then explored the use of selective primers to achieve a greater complexity reduction during GBS library preparation. The number of SNP calls could be increased by almost 40% and their depth of coverage was more than doubled, thus opening the door to an increase in the throughput and a significant decrease in the per sample cost. The approach to obtain high quality SNPs developed here will be helpful for marker assisted genomics as well as assessment of available genetic resources for effective utilisation in a wide number of species.

## Introduction

Molecular markers are extremely useful in plant as well as animal genetics and genomics. Markers are prerequisite for mapping and tagging of genes/quantitative trait loci (QTLs), segregation analysis, genetic diagnosis, forensic examination, phylogenetic analysis, and numerous molecular biology applications [1]–[4]. Several types of molecular markers have been developed and are routinely being used in molecular biology labs. However, most of these marker systems are constrained in their use because of their limited availability and/or the high cost of analyses conducted on a large scale. Among the various types of markers in use (see reviews by Agarwal et al. and Sonah et al.) [5], [6], single nucleotide polymorphisms (SNPs) are the most abundant in a genome and those are suitable for analysis on a wide range of scales [7], [8]. However, the development of high-throughput genotyping platforms for large numbers of SNPs (thousands to millions) has proved relatively lengthy and costly. Typically, a fairly large sequencing effort is devoted to identifying polymorphic sites in a genome among a set of lines deemed of interest. Then, a subset of these SNPs is selected (based on their position in the genome and their suitability for the assay of choice) to develop a genotyping platform capable of assaying all chosen SNPs in parallel. For instance, in a major crop such as soybean, we have seen the recent development of such platforms like the Universal Soybean Linkage Panel (USLP 1.0), which allows the simultaneous interrogation of 1,536 SNP loci through a GoldenGate assay [9]. More recently, Haun et al. developed an Infinium assay capable of testing 44,000 SNP loci [10]. On such assays, the number of SNPs that prove informative within a given set of lines will typically range between 1/3 (narrow set) and 2/3 (diverse set).

High-throughput next generation sequencing (NGS) technologies have opened the way to novel approaches in this area because of their capacity to produce sequence information on an unparalleled scale compared to Sanger sequencing [11]. In soybean, re-sequencing of 31 genotypes identified a set of 205,614 SNPs that provide a valuable genomic resource [3]. However, such re-sequencing efforts are not yet suitable as a SNP genotyping approach, as this would prove too costly to carry out on a large set of genotypes. To reduce the cost without compromising quality of the SNPs, several methods have been developed that involve sequencing only a small fraction of the entire genome. Three main complexity reduction methods, namely Reduced Representation Libraries (RRL), Restriction site Associated DNA (RAD) sequencing, and Genotyping By Sequencing (GBS) have been described to date (reviewed in Davey et al.) [12] Using the RRL approach in soybean, 14,550 and 25,047 SNPs were identified by two independent studies [13], [14]. Although highly promising as a SNP discovery tool, such an RRL approach is not very practical as a genotyping tool for use on a large scale, as the amount of DNA used in this work ranged between 10 and 50 µg. More recently, Elshire et al. working with maize and barley, described a GBS method that offers a greatly simplified library production procedure more amenable to use on large numbers of individuals/lines [15]. In the absence of a size selection step on the digested DNAs (as is necessary in the RRL approach), it can be carried out using small amounts of DNA (100 ng). Poland et al. have extended this work by exploring a two-enzyme GBS protocol (*Pst*I/*Msp*I) that provides a greater degree of complexity reduction than the original protocol using *Ape*KI [16].

In the present investigation, we demonstrate the effective use of the *Ape*KI enzyme for GBS library preparation in soybean and describe a novel pipeline for calling SNPs starting from the sequence reads. Following an in-depth characterization of the SNPs obtained via this pipeline, we describe a modified library preparation protocol in which selective amplification is used to increase both the number of SNPs called and their depth of coverage. The resulting increase in efficiency is shown to allow an important reduction in per sample cost.

## Materials and Methods

### Plant Material

A set of eight diverse soybean genotypes (Set A) of different origins and maturity groups (MG) was used for the identification of SNPs. The set included three cultivars from eastern Canada (Maple Donovan, Toma and PS46RR; MGs 0, 00 and 0, respectively), two cultivars from the United States (S19-90 and Williams 82; MGs I and II, respectively), as well as two genotypes from Africa (TGx1989-53F and TGx1990-67F; MG IV), and one from Brazil (Ocepara-4; MG IV). Williams 82 has been completely sequenced and was used as a reference. A second set of eight genotypes from Canada (Set B; QS4003.28B, OAC Thames, OAC Eramosa, OAC09-01C, AC Harmony, PI159925, X5331-1-S1-1S-3-B, X5194-1-54-2-1-B) were used for SNP validation by Sanger sequencing along with Set A. Finally, Maple Donovan and OAC Bayfield were used for the optimization of GBS using primers with selective bases.

### DNA Extraction, Library Preparation and Sequencing

DNA was extracted from 100 mg fresh young leaves using the DNeasy 96 Plant kit (Qiagen, cat. no. 69181) following the manufacturer’s protocol. DNA was quantified using Quant-iT™ PicoGreen® as well as Thermo Scientific Nanodrop 8000 spectrophotometer instrument (Fisher Scientific). DNA concentrations were normalized to 10 ng/µl and subsequently used for library preparation. Sequencing libraries were prepared according to the GBS protocol as per Elshire et al. except for the use of selective primers described below [15]. Single-end sequencing was performed on a single lane of an Illumina Genome Analyzer II (at the McGill University-Génome Québec Innovation Center in Montreal, Canada) for the initial development of the method using a 48-plex GBS library of which 8 barcodes were devoted to soybean Set A. Subsequent work was performed on an Illumina HiSeq2000.

### Scalable Complexity Reduction

A scalable complexity reduction was achieved by using longer 3′ primers that cover the entire common adapter, the 3′ restriction site and extend 1 or 2 bases into the insert (5′-CAAGCAGA-AGACGGCATACGAGATCGGTCTCGGCATTCCTGCTGAACCGCTCTTCCGATCTCAGCXY-3′). During the PCR amplification step performed on pooled adapter-ligated restriction fragments, 18 cycles were performed with such a modified 3′ primer and the standard 5′ primer (5′-AATGATACGGCGACCACCGAGATCTACACTCTTTCCCTACACGACGC-TCTTCCGATCT-3′).

### *In silico* Assessment of Restriction Enzymes, Genes and Transposable Elements

*In silico* digestion of the soybean genome sequence (http://phytozome.net) was performed for different restriction enzymes. The resultant fragments were localized on pseudo-molecules and the frequency distribution was calculated for each chromosome. The frequency distribution of fragment size was also evaluated to determine the proportion of fragments in the preferred size range (100–400 bp). In addition, the frequency of predicted protein coding genes and repetitive elements like *Gypsy*, *Copia* and other minor classes of transposons were calculated. The information of transposable elements was retrieved from a soybean transposon database ‘soytedb’ (http://soybase.org) and co-localized with SNPs and predicted genes on the soybean genome.

### Processing of Illumina Raw Sequence Read Data and SNP Calling

**Figure 1:**
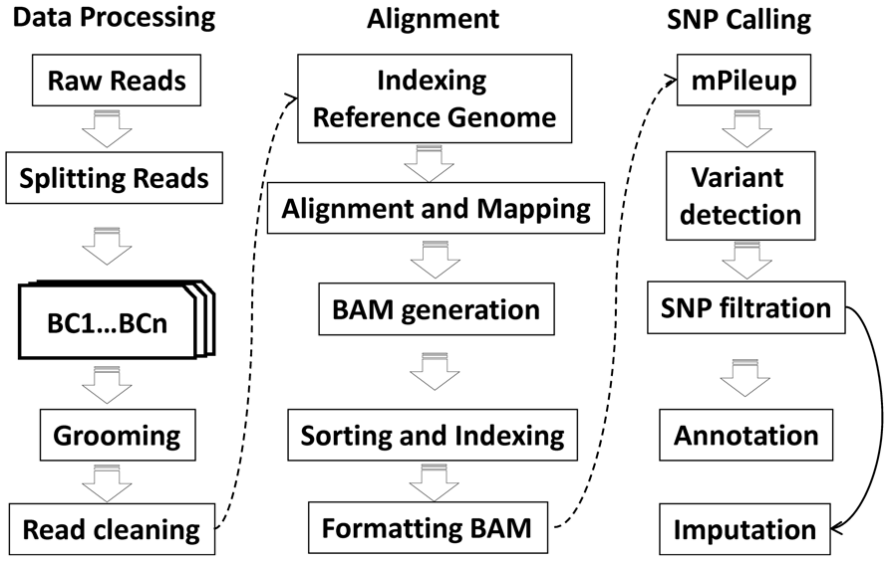
Flowchart showing steps performed for the identification of SNPs in the IGST-GBS pipeline. The process can be divided into three main steps: data processing, mapping and SNP calling.

A pipeline implemented in perl programming language was developed for the processing of Illumina sequence read data (IGST-GBS pipeline; J. Laroche, unpublished). The steps involved in the pipeline were executed in separate shell scripts. This pipeline uses different publicly available software tools (FASTX toolkit, BWA, SAMtools, VCFtools) as well as some in-house tools and a flow diagram of the process is presented in Figure 1. The raw SNPs obtained were further filtered using VCFtools based on read depth, missing data in genotypes and minor allele frequency. Heterozygous correction was performed by an in-house python script. Finally, missing information in filtered set of quality SNPs were imputed using fastPHASE software [17].

### Experimental Validation of SNPs

A subset of 24 randomly selected SNPs identified using the IGST-GBS pipeline was used for validation by Sanger sequencing. Primers were designed to obtain amplicons of 500–1,000 bases containing at least one putative SNP. PCR reactions of 10 µl volume containing 20 ng of genomic DNA, 0.5 µM each of forward and reverse primers, 0.1 mM dNTPs, 1x PCR buffer and 0.2 unit of Taq DNA polymerase was performed in a thermal cycler. The cycling conditions involved initial denaturation at 94°C for 4 min, followed by 35 cycles of denaturation at 94°C for 1 min, primer annealing at 55–60°C for 1 min, and primer extension at 72°C for 1 min. A final extension at 72°C for 7 min was performed and products stored at 4°C until electrophoresis. The PCR products were resolved by electrophoresis in 2% agarose gels in 1x TBE buffer and visualized by ethidium bromide staining. The PCR amplicons were sequenced on an Applied Biosystems 3130XL at the Plateforme d’analyses génomiques (IBIS, Université Laval). All the sequences along with reference sequence were assembled using the SeqMan tool available in DNAstar software package (www.dnastar.com).

### Functional Annotation of SNPs

Soybean genome annotation information in GFF3 format was retrieved from phytozome (www.phytozome.com). The soybean genome annotation provided predicted gene structure and verified exon/intron boundaries using the ESTs and cDNA data. Therefore we have co-located all the SNPs with gene models and predicted their structural and functional relevance in the genome. A SnpEff v3.0 open source program was also used for variant annotation and effect prediction of SNPs (http://snpeff.sourceforge.net/) [18]. SNPs were described on the basis of their structural occurrence in the intergenic region, exons, introns, 5` UTR, 3`UTR, or exon-intron splicing sites. Moreover, functional relevance (synonymous, nonsynonymous) of the SNPs was determined.

### Phylogenetic Analysis

SNP data for the eight diverse soybean cultivars were used to generate a phylogenetic tree. The matrix of genetic distances between the cultivars was obtained using the PHYLIP 3.69 package (http://evolution.gs.washington.edu/phylip.html). Subsequently, a consensus neighbour-joining tree was constructed after 1,000 bootstrap replications using PHYLIP and a graphical representation of the resulting tree was produced using MEGA5 (www.megasoftware.net).

## Results

### Selection of an Appropriate Enzyme for GBS in Soybean

**Figure 2:**
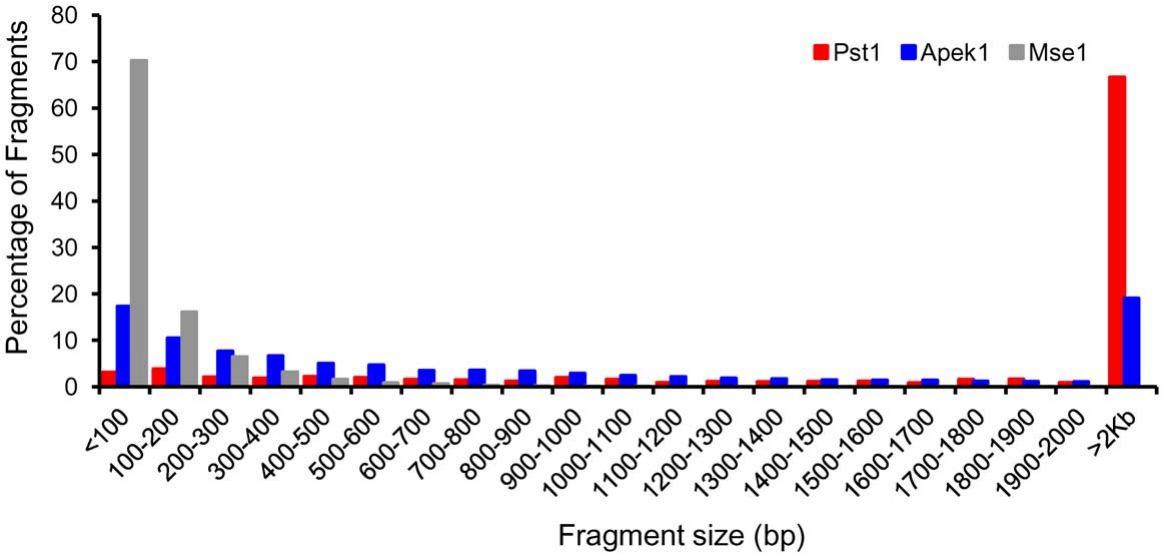
*In silico* analysis of restriction enzyme sites in the soybean genome. Fragment size distribution obtained by *in silico* digestion of soybean chromosome 5 with *Ape*K1, *Pst*1 and *Mse*1 restriction enzymes showing a higher percentage of *Ape*K1 fragments in a suitable range for genotyping by sequencing.

Choosing the appropriate restriction enzyme is a critical step in developing a GBS protocol for an organism. In the absence of a size selection step during library preparation, it is important to maximize the proportion of predicted restriction fragments that fall within the desired size range (100–400 bp) for sequencing. We performed *in silico* digestion of the soybean genome with three restriction enzymes (*Mse*I, *Ape*KI and *Pst*I) previously used in soybean and other plant species. We evaluated the number of expected restriction sites and the size distribution of predicted fragments. *Mse*I was predicted to generate up to 9.5 million fragments, *Ape*KI 800 K fragments and *Pst*1 100 K fragments in the soybean genome. These represent maximal values, as methylation would be expected to impede digestion at some sites. Nonetheless, these large differences in the number of restriction sites translated into marked differences in the expected distribution of fragment sizes. As illustrated in Figure 2, the largest fraction of fragments expected to be in the desired range was predicted following digestion with *Ape*KI. *Mse*I produced a large majority of very small fragments not expected to perform well on the Illumina sequencing platform during bridge amplification. *Pst*I, even if used in combination with a frequent cutter such as *Msp*I in a two-enzyme strategy, imposed a fairly low ceiling on the number of genomic regions potentially interrogated (<100 K).

### Sequencing and Mapping

**Figure 3:**
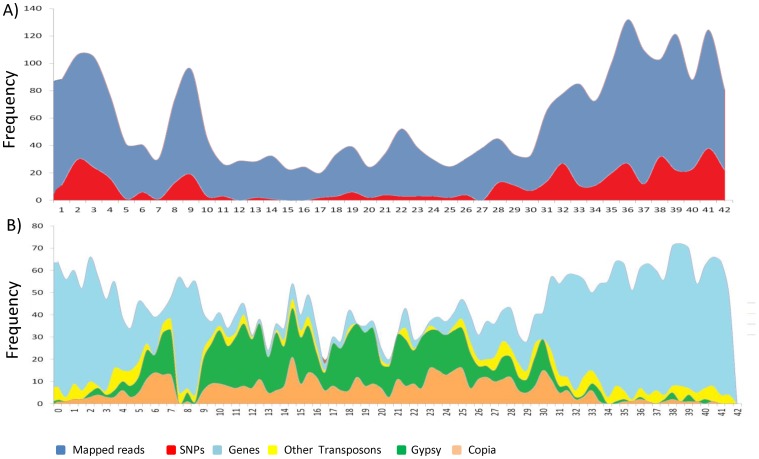
Sequence coverage and SNP distribution. (a) Distribution of mapped sequence reads (scaled down to 1/10) and SNPs identified using a GBS approach, and (b) corresponding frequency of genes and transposons identified in the same bins on soybean chromosome 5. All the transposons and genes were retrieved from the soybase and phytozome database respectively (www.soybase.org, www.phytozome.org).

Eight diverse soybean genotypes (Set A) were used in a 48-plex *Ape*KI library that included DNA from five other species of plants and animals. A total of 5.54 million raw sequence reads were obtained by single-end sequencing of the eight soybean genotypes on an Illumina Genome Analyzer II (Table 1). Sorting of raw reads was performed using the barcode information associated with each read. The number of sorted raw sequence reads ranged from 0.44 million reads (TGx1989-53F) up to 1.00 million reads (Ocepara-4). The quality of individual reads was assured by checking the proper read layout consisting of a barcode followed by a restriction site. All the steps in read processing were performed by our pipeline and these included grooming, barcode splitting, trimming, and adapter clipping (Figure 1). In addition, poor quality sequence reads along with reads shorter than 25 bases or reads containing “Ns” were discarded. Finally, a total of 5.50 million processed quality reads (98.76% of all reads) were retained, a proportion that was highly uniform in all the genotypes (Table 1). Processed reads of the individual genotypes were mapped onto the reference genome and only reads mapping to a unique location in the genome were retained. Overall, such uniquely mapped reads represented 85% of the total and were well distributed across the chromosomes (Figure 3). An analysis of unmapped reads revealed that chloroplast and mitochondrial DNA contributed approximately 4.6% and 1.8% of the total population of reads. Many of the remaining unmapped reads showed similarity with repetitive sequence elements like transposons or satellite DNA.

**Table 1:** Summary of sequenced raw and processed reads in eight soybean genotypes obtained on an Illumina Genome Analyzer II.

Genotypes	Maple Donovan	Toma	S19-90	Williams 82	PS46RR	TGx1989-53F	TGx1990-67F	Ocepara-4		Total
**Raw Reads**	540,827	805,460	763,541	877,607	578,458	440,636	526,300	1,003,014	5,535,843	
**Processed Reads (%)**	98.77	98.75	98.77	98.77	98.76	98.76	98.76	98.76	98.76	
**Mapped Reads (%)**	82.58	85.58	84.64	86.96	85.23	83.47	85.60	84.64	85.00	

### SNP Discovery and Distribution

Using the IGST-GBS pipeline that we have developed, a total of 33,553 polymorphisms (SNPs and indels) were identified, with indels representing 5% of the total. Subsequently, filtering of SNPs on the basis of read depth (minimum of two reads/SNP/individual) yielded a total of 10,120 high quality SNPs. The average depth of coverage was 7.8 reads/SNP locus, thus ensuring a low level of missing data (8%). Among the called genotypes, the vast majority were homozygous for the reference (53.3%) or an alternate allele (33.6%), whereas 4.9% were heterozygous and 8.1% were missing.

The frequency of SNPs on the twenty soybean chromosomes averaged 10 SNPs/Mb. The distribution of SNPs was similar for all chromosomes and is illustrated for chromosome 5 in Figure 3a. In comparing this distribution with those of genic and repetitive sequences for the same chromosome (Figure 3b), it is clear that the distribution of SNPs closely mirrored the distribution of genic sequences; it proved to be highest in gene-rich terminal regions and lowest in highly repetitive centromeric and pericentromeric regions of chromosomes. The highest number of SNPs was identified on chromosome 18 (836 SNPs) followed by chromosome 15 (716 SNPs) and the lowest numbers of SNPs were observed on chromosome 12 (273 SNPs) followed by 286 SNPs on chromosome 11 (Figure S1). The number of SNPs identified on each chromosome was significantly correlated (r^2^ = 0.36) with the physical length of chromosome.

### Experimental Validation of SNPs

A set of 24 randomly selected SNP loci identified based on the standard GBS library preparation protocol were amplified in 16 genotypes (sets A and B) and sequenced by Sanger sequencing. The PCR amplification of all the loci showed a single sharp band after agarose gel electrophoresis (Figure S2a). Subsequent sequencing of the PCR amplicons confirmed the presence of almost all the SNPs (Table 2, Figure S2b). Out of 384 (24×16) individual genotypes, 376 (98%) were successfully validated by Sanger sequencing, thus confirming the high quality of this filtered set of SNPs (Table 2). In most cases of disagreement between the GBS and Sanger genotype calls (6 out of a total of 8), the contentious genotype was called as a heterozygote in the IGST-GBS pipeline. As a third of all heterozygous genotype calls were not confirmed by Sanger sequencing (6/18), we systematically removed all such calls from the dataset. As for indels, only four could be verified among the 24 amplicons, and these were confirmed in all cases.

**Table 2:** Validation of single nucleotide polymorphism calls by Sanger sequencing.

Genotypes	Concordant			Discordant			Validation (%)
	AA	AB	BB	AA	AB	BB	
Set A	115	3	71	0	1	2	98.4
Set B	111	9	67	0	5	0	97.4

Set A = Maple Donovan, Toma, S19-90, Williams 82, PS46RR, TGx1989-53F, TGx1990-67F, Ocepara-4.

Set B = QS4003.28B, OAC Thames, OAC Eramosa, OAC09-01C, AC Harmony, PI159925, X5331-1-S1-1S-3-B, X5194-1-54-2-1-B.

AA-homozygous for reference allele, AB-heterozygous, BB-homozygous for alternate allele.

### Structural, Functional and Evolutionary Impact of SNPs

**Figure 4:**
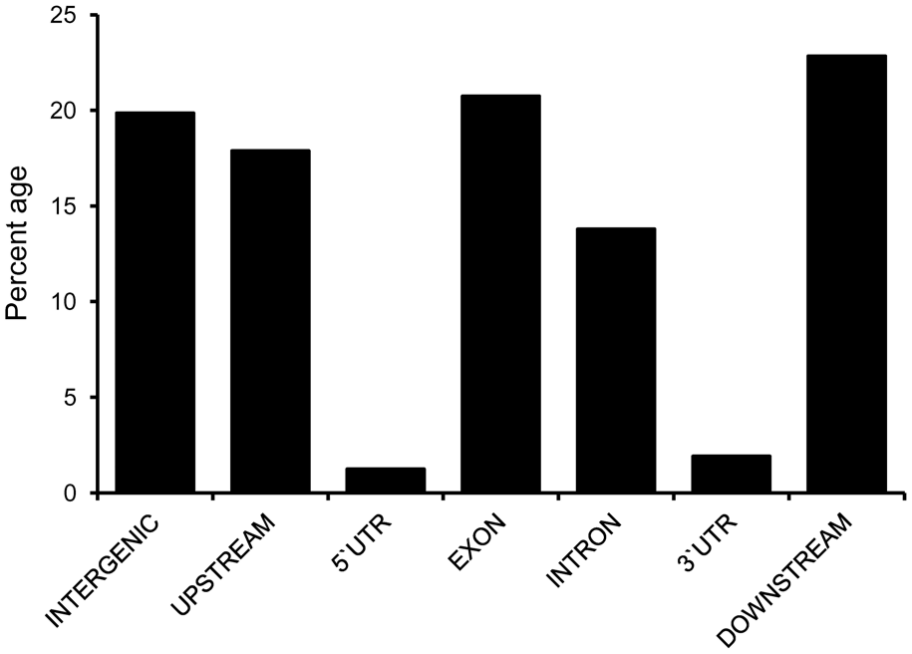
Distribution of SNPs on the basis of their location in respective predicted gene models in soybean genome. SNPs were categorised using gene structure information retrieved from phytozome (www.phytozome.org).

The structural and functional relevance of SNPs were investigated by comparing the location of the 10,120 SNPs with the coordinates of predicted gene structure. It revealed that 60.57% of these SNPs resided in intergenic regions, of which 17.88% were within 5 kb immediately upstream and 22.83% within 5 kb immediately downstream of an open reading frame (ORF) (Figure 4). The remaining SNPs (39.43%) were thus situated in exons, introns, or untranslated regions (UTRs). A higher percentage of SNPs was observed in exonic regions (20.73%) than in introns (13.78%), 5′ UTRs (1.91%) or 3′ UTRs (1.24%). In addition, nine SNPs were observed at intron splicing sites which could potentially alter the function of these genes. In a further analysis, SNPs located in the coding region were categorized as synonymous or non-synonymous. We found that about half (51%) of the observed SNPs would result in a change of amino acid or introduction of a stop codon whereas the remaining half (49%) were silent mutations (Table S1).

### Phylogenetic and Pairwise Diversity Analysis

**Figure 5:**
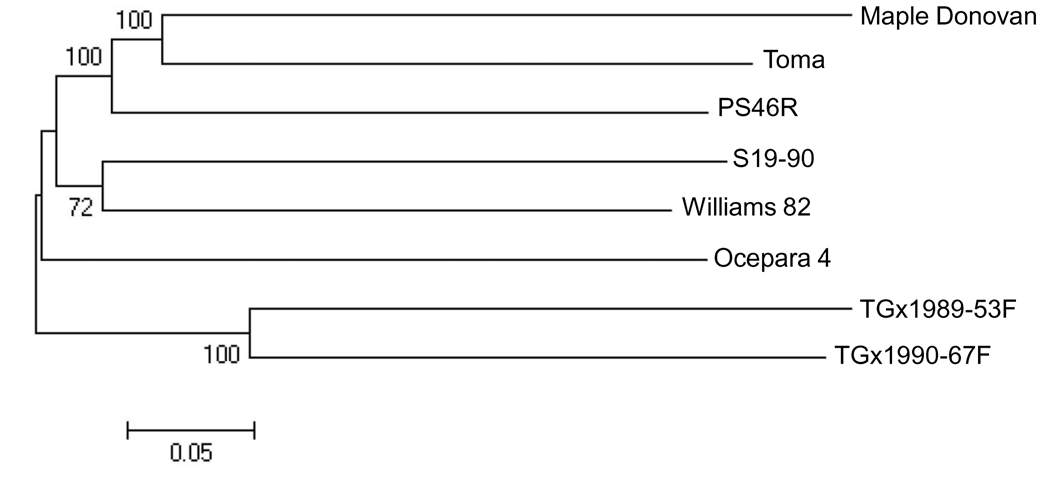
Phylogenetic tree showing genetic distance among a set of eight diverse soybean cultivars. The phylogenetic tree was constructed on the basis of 10,120 SNPs identified using the GBS approach.

As a further genome-wide validation of the quality of the data, we produced a phylogenetic tree of the eight genotypes of set A with the full set of 10,120 SNPs (Figure 5). The resulting tree contains four well-resolved clades grouping genotypes in accordance with their distinct geographic origins and maturity groups (Figure 5). The African genotypes were found as most diverse from other genotypes and the genotypes from the United States and Canada grouped closer.

Analysis of pairwise SNP polymorphism (Table S2) showed that the largest number of informative SNPs was observed between Maple Donovan and TGx1989-53F (5,807 SNPs) and the lowest number of informative SNPs was observed between TGx1990-67F and TGx1989-53F (4,058 SNPs).

### Optimizing the Number and Coverage of SNPs by the Use of Selective Primers

**Figure 6:**
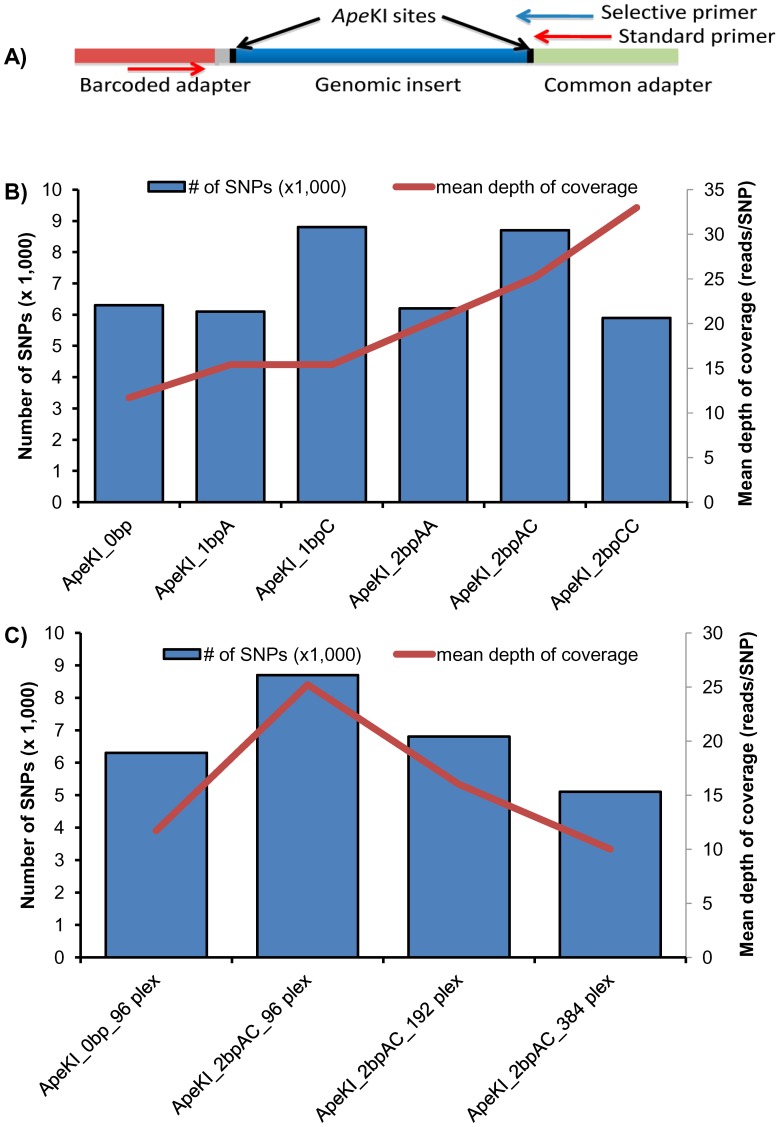
Impact of selective amplification on the number and depth of coverage of SNPs. (a) Schematic representation of an *Ape*KI restriction fragment flanked by suitable ligated adapters and the position of standard or selective primers, (b) Comparison of the number of SNPs and sequence read depth obtained with different sets of primers, and (c) number of SNPs and mean depth of coverage observed with selective amplification of *Ape*KI digested fragments with AC selective primers at different levels of multiplexing.

In examining the depth of coverage of the reads for each individual, we noticed that a large proportion of the reads (∼40%) were unique, i.e. they were seen only once. As our pipeline required a mean coverage of two reads per locus per individual for a SNP to be called with high confidence, we hypothesized that it might be preferable to amplify only a subset of all possible *Ape*KI restriction fragments. This selectively amplified subset would present a higher mean depth of coverage and a higher percentage of the interrogated loci would lead to a successful genotype call. We therefore produced GBS libraries in which the final amplification step was performed with a selective primer extending across the 3′-*Ape*KI site and 1 or 2 bases into the insert (Figure 6a).

Using the same number of reads (2 M) obtained for each of two genotypes (Maple Donovan and OAC Bayfield) following library construction with a common primer having 0 (i.e. the standard *Ape*KI protocol), 1 (A or C) or 2 (AA, AC or CC) selective bases at the 3′ end, we obtained a significant improvement in both the number and the depth of coverage of called SNPs. As can be seen in Figure 6b, most libraries prepared using selective amplification resulted in a greater number of SNP calls with an improved depth of coverage. Among the tested combinations, a common primer with two selective bases (AC) resulted in a 38.4% increase in the number of called SNPs (8,652 vs 6,252) all the while more than doubling the mean depth of coverage (25.2 vs 11.7 reads/locus).

The resulting increase (over 2-fold) in depth of coverage suggested that it should be possible to increase the degree of multiplexing without seeing a concomitant decrease in the number of SNP calls. To simulate such an increase in multiplexing, we reduced the number of reads per sample from 2 M to 1 M and then 0.5 M. Assuming that 200 M reads can be obtained per lane on the Illumina HiSeq, these numbers correspond to the average number of reads/sample expected for 96-, 192- and 384-plex libraries. As can be seen in Figure 6c, doubling the degree of multiplexing still resulted in more called SNPs (6,846 vs 6,252) and greater depth of coverage (16.0 vs 11.7) than the standard *Ape*KI protocol (0 selective base). At 384-plex, a slight decrease in the number of called SNPs (5,082) and depth of coverage (10.0) was suffered in exchange for a 4-fold increase in the throughput of the analysis.

## Discussion

The simultaneous identification and genotyping of SNPs is now possible because of the important recent advances in sequencing [12]. Among the three current methodologies (RRL, RAD and GBS) being used via NGS for SNP genotyping, GBS provides many advantages. It offers a much simplified library preparation procedure that can be performed with small amounts of starting DNA (100–200 ng) and is amenable to a high level of multiplexing. Also, following a judicious choice of enzyme, it can provide a high SNP coverage in gene-rich regions of the genome in a highly cost-effective manner. The original GBS protocol developed in maize and barley demonstrated the advantages of such an approach [15].

The availability of an excellent reference genome for soybean provided us with a solid basis for the selection of a good restriction enzyme for GBS in this crop [19]. A uniform distribution of the *Ape*K1 restriction sites was observed following *in silico* digestion of the soybean genome and, more importantly, a good proportion of the resultant fragments were short enough for effective amplification and sequencing on the Illumina platform (Figure 2). However, as *Ape*K1 is partially sensitive to methylation, the resultant fragments achieved in a wet lab may vary from the *in silico* prediction. Nonetheless, we show in this work that the number of SNPs obtained with *Ape*KI is very similar to the numbers obtained in previous RRL work in soybean [14], all the while requiring much-reduced amounts of DNA. Furthermore, the sensitivity to methylation provides the advantage of more extensive cutting in the less methylated, single-copy gene-rich regions of the genome. This proved to be the case in soybean, where the distribution of SNP markers was distinctly skewed in favour of gene-rich regions. This seemed in contrast to the situation initially described in barley and wheat using a two-enzyme protocol (*Pst*I/*Msp*I), as the highest density of SNPs had been reported in the centromeric and pericentromeric regions [16]. However, in the recently published draft genome of barley, it has been shown that the density of SNPs detected through a GBS approach largely follows gene density [20]. In the current work, we made two important and widely-applicable contributions to the GBS approach in addition to developing an optimized protocol for performing GBS.

### Improved Versatility of the GBS Approach

Our first innovation regards the GBS library preparation process. We demonstrate how selective primers can be used to amplify and sequence only a subset of the restriction fragments obtained following digestion. As anticipated, this results in an increase in the depth of coverage of the SNPs as a given sequencing effort is focused on a smaller set of amplicons. Somewhat counterintuitive, however, is the observed increase in the number of SNP calls using certain selective primers. As was explained above, we hypothesize that this as a consequence of the large pool of restriction fragments that are sequenced only once and that do not offer sufficient depth of coverage to meet our criteria for the calling of high-quality SNPs with the regular protocol. An immediate benefit of the new protocol is that a greater level of multiplexing can be supported without any reduction in the number or depth of coverage of SNPs. We have demonstrated that, using a primer with 2 selective bases (AC), a 2-fold increase in the level of multiplexing still results in an increased number of SNPs (+9.5%) and depth of coverage (+37%). Also, even after increasing 4-fold the number of lines analyzed, the number of SNPs obtained is reduced by less than 20% (5,082/6,252) while depth of coverage is reduced by less than 15% (10.0/11.7).

If we follow the same logic, one can anticipate that using primers with even more selective bases will continue to increase the depth of coverage of the SNPs but will eventually also lead to a decrease in the number of called SNPs. This has some important implications for the versatility of use of a given enzyme used for making GBS libraries. If using a range of selective primers allows one to adjust at will the number of SNPs obtained, a single enzyme with suitable distribution in the genome (such as *Ape*KI for soybean) could be amenable for a wider range of applications. For example, in cases where a lower SNP density is sufficient (such as a biparental mapping population or characterizing genetic diversity in broad sets of germplasm), one could use the appropriate primers to obtain the desired number of SNPs instead of resorting to a different, less frequent-cutting enzyme with all the required sets of barcoded adapters and primers that this would call for. The resulting increase in the depth of coverage would allow for an increase in the multiplexing of the libraries, without reducing the number of SNPs that can be called successfully, thus reducing the per-sample cost.

As described above, there are important benefits to using a modified GBS library preparation protocol in which primers with selective bases are used. In soybean, the use of two selective bases in such primers always resulted in an increased depth of coverage of the SNPs, but not always an increase in the number of SNPs (compare AA, AC and CC in Figure 6b). This presumably reflects the frequency of dinucleotides flanking *Ape*KI sites and the stringency of amplification resulting from the use of such primers. This will likely vary from species to species and need to be discovered empirically, but is very simple to optimize.

### An Alternative and Flexible Pipeline for Calling SNPs

Another contribution made to GBS is an alternative analytical pipeline assembled using widely available tools that produce SNP catalogs in information-rich variant call format (.vcf) files. Although the GBS analysis pipeline developed by the Cornell team and integrated in TASSEL uses a similar overall approach, it differs in some important respects. Firstly, in the TASSEL pipeline, the length of the reads used to call SNPs is limited to a maximum of 64 bases. Although this reduces the computation time, it also reduces the potential to identify SNPs in a read, as the average length of our reads after removal of the barcode and adapter sequence was ∼80 bases. Secondly, the output of our pipeline is in variant call format (.vcf), one that is widely used in SNP data analysis and on which a broad range of filtration tools can be applied, for example using VCFtools. Depending on the skill of the user, there are lots of options to modify the SNP calling parameters. In contrast, in the Hapmap format produced by the TASSEL pipeline, there are fewer possibilities for a user to modify the stringency of the SNP calling aspect of the pipeline. In side-by-side comparisons (data not shown), both pipelines produced a very similar tally of SNPs and a majority of these (57%) were called in identical fashion. An examination of the differences in SNP calls between the two pipelines suggests that most of the SNPs that are called by the IGST pipeline and not the TASSEL pipeline reside in the bases beyond the 64-base limit imposed in the TASSEL pipeline. As for SNPs called by the TASSEL pipeline and not the IGST pipeline, these were mostly due to a more permissive set of SNP calling parameters.

Despite the fact that the IGST pipeline uses stringent SNP calling parameters, as many as a third of the heterozygous calls were not validated by Sanger sequencing. As it is probable that such erroneous calls could be due to sequence divergence at paralogous loci, we systematically removed these so as not to introduce incorrect data.

We conclude that the improved GBS protocol described in this work allows one to identify large numbers of high quality SNPs in soybean in a very cost-effective manner. The improvements we propose to the GBS library making protocol could be applied to any species of interest, starting from a protocol anchored on a suitable enzyme. Similarly, the IGST-GBS pipeline is suited to the analysis of any species for which a reference genome is available. As it offers a wide suite of options for filtering SNPs, it can be optimized for various types of populations and analyses.

## Supporting Information

Figure S1 **Frequency distribution of SNPs identified in eight soybean cultivars using the GBS approach.** Frequency was calculated for each 1 Mb bin on all 20 soybean chromosomes
(TIF)

Figure S2 **SNP confirmed using re-sequencing of loci using Sanger sequencing method.** a) Agarose gel showing PCR amplification of SNP loci in different soybean cultivars. b) Sequence alignment showing SNP alleles in soybean cultivars.
(TIF)

Table S1 **Functional annotation of SNPs.** Annotation was performed using snpEff v.3 for a collection of SNPS obtained by Illumina sequencing of a GBS library prepared from eight soybean genotypes.
(XLSX)

Table S2 **Number of SNPs between pairs of soybean genotypes.**
(DOCX)
