# Supplementary figures and images for: An Improved Genotyping by Sequencing (GBS) Approach Offering Increased Versatility and Efficiency of SNP Discovery and Genotyping
Source: PLoS One. 2013 Jan 23;8(1):e54603. doi: 10.1371/journal.pone.0054603 (PMC3553054; doi:10.1371/journal.pone.0054603)

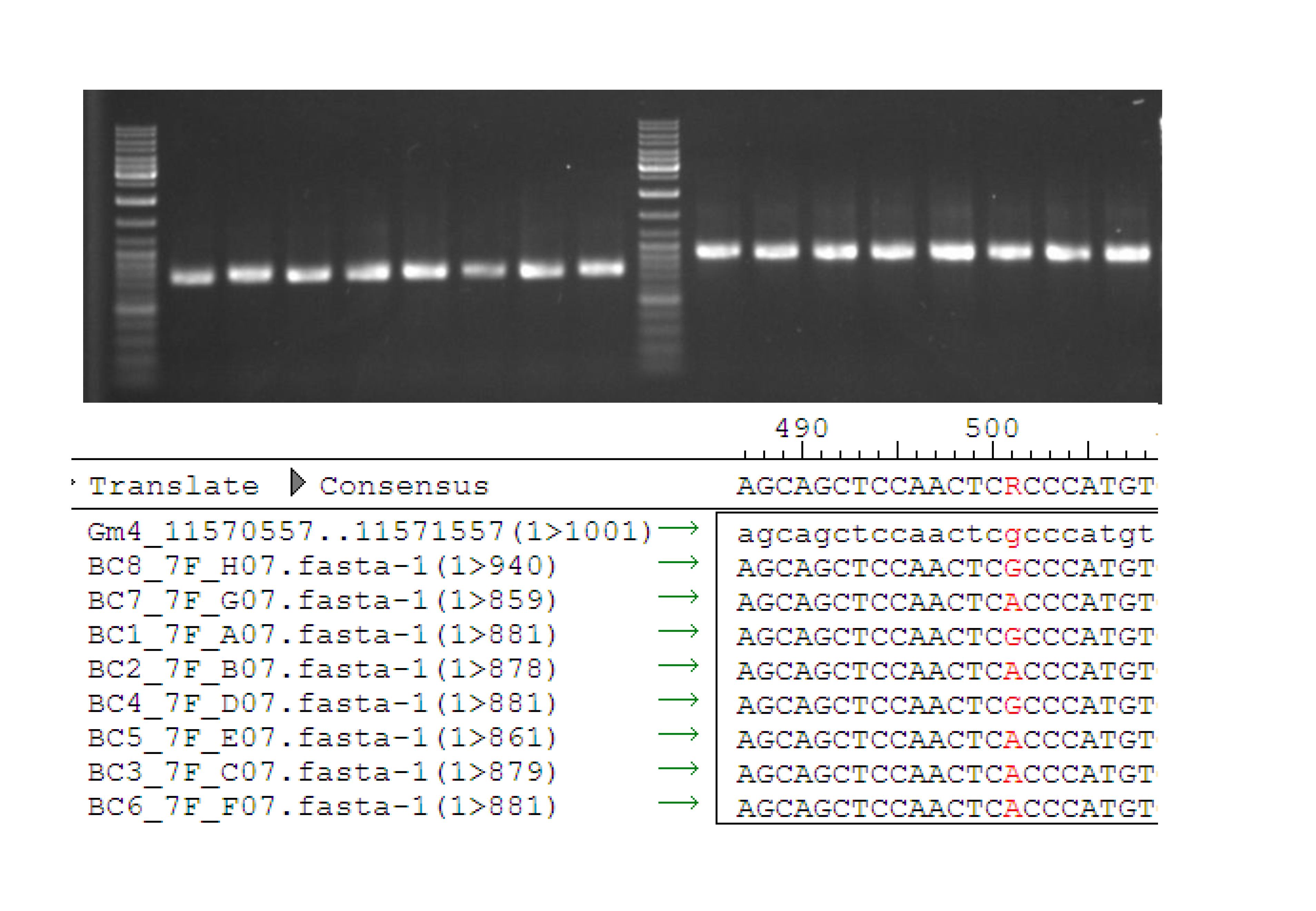

Supplement: Figure S2 — SNP confirmed using re-sequencing of loci using Sanger sequencing method. a) Agarose gel showing PCR amplification of SNP loci in different soybean cultivars. b) Sequence alignment showing SNP alleles in soybean cultivars. (TIF) [file pone.0054603.s002.tif]
