# Supplementary material for: An Improved Genotyping by Sequencing (GBS) Approach Offering Increased Versatility and Efficiency of SNP Discovery and Genotyping
Source: PLoS One. 2013 Jan 23;8(1):e54603. doi: 10.1371/journal.pone.0054603 (PMC3553054; doi:10.1371/journal.pone.0054603)
Supplement: Table S2 — Number of SNPs between pairs of soybean genotypes. (DOCX) [file pone.0054603.s004.docx]

**Table S2. Number of SNPs between pairs of soybean genotypes.**

| **Genotype** | **Maple Donovan** | **Toma** | **S19-90** | **Williams 82** | **PS46RR** | **TGx1989-53F** | **TGx1990-67F** | **Ocepara-4** |
| --- | --- | --- | --- | --- | --- | --- | --- | --- |
| **Maple Donovan** | - |  |  |  |  |  |  |  |
| **Toma** | 4287 |  |  |  |  |  |  |  |
| **S19-90** | 5285 | 4642 |  |  |  |  |  |  |
| **Williams 82** | 5077 | 5379 | 4228 |  |  |  |  |  |
| **PS46RR** | 4914 | 4205 | 4425 | 4636 |  |  |  |  |
| **TGx1989-53F** | 5807 | 5676 | 5531 | 5152 | 5609 |  |  |  |
| **TGx1990-67F** | 5785 | 5551 | 5457 | 5460 | 5377 | 4058 |  |  |
| **Ocepara-4** | 5311 | 5063 | 5108 | 4547 | 5400 | 5400 | 5187 | - |
